# Hydrological, Environmental and Taxonomical Heterogeneity during the Transition from Drying to Flowing Conditions in a Mediterranean Intermittent River

**DOI:** 10.3390/biology10040316

**Published:** 2021-04-09

**Authors:** Andy Banegas-Medina, Isis-Yelena Montes, Ourania Tzoraki, Luc Brendonck, Tom Pinceel, Gustavo Diaz, Pedro Arriagada, Jose-Luis Arumi, Pablo Pedreros, Ricardo Figueroa

**Affiliations:** 1 Centre of Environmental Sciences EULA-Chile and CHRIAM Water Research Centre, Department of Aquatic Systems, Faculty of Environmental Sciences, Universidad de Concepción, P.O. Box 160-C Concepción, Chile; imontes@udec.cl (I.-Y.M.); gusdiaz@udec.cl (G.D.); papedrer@udec.cl (P.P.); rfiguero@udec.cl (R.F.); 2 Laboratory of Biology, Department of Sciences, Danlí Technological Campus, Universidad Nacional Autónoma de Honduras, Panamerican Highway km 95, 13201 Danlí, Honduras; 3 Marine Sciences Department, School of Environment, University of the Aegean, University Hill, 81100 Mytilene, Lesvos, Greece; rania.tzoraki@aegean.gr; 4 Laboratory of Animal Ecology, Global Change and Sustainable Development, KU Leuven, Ch. Deberiotstraat 32, 3000 Leuven, Belgium; luc.brendonck@kuleuven.be (L.B.); tom.pinceel@kuleuven.be (T.P.); 5 Water Research Group, Unit for Environmental Sciences and Management, North-West University, Private Bag X6001, 2520 Potchefstroom, South Africa; 6 Centre for Environmental Management, University of the Free State, P.O. Box 339, 9300 Bloemfontein, South Africa; 7 Centre of Environmental Sciences EULA-Chile and CHRIAM Water Research Centre, Department of Environmental Engineering, Faculty of Environmental Sciences, Universidad de Concepción, P.O. Box 160-C Concepción, Chile; parriagada@udec.cl; 8 Water Resources Department, Faculty of Agricultural Engineering and CHRIAM Water Research Centre, Universidad de Concepción, Vicente Mendez 595, 3812120 Chillán, Chile; jarumi@udec.cl

**Keywords:** intermittent rivers and ephemeral streams, temporary rivers, TREHS Tool, aquatic invertebrates, drying, rewetting, disconnected pools

## Abstract

**Simple Summary:**

In recent decades, the riverine ecosystems have been considered to evaluate the aquatic biological diversity, hydrological variations, and ecosystem services. However, climatic change scenarios and anthropogenic interventions are expected to shift from perennial to intermittent rivers with possible repercussion on aquatic biodiversity and human well-being. Our study identified a significant reduction in the Mediterranean intermittent river streamflow with an increase of zero flow days in the last decades. Furthermore, the aquatic invertebrates showed variations during the transition from drying to rewetting with a significantly changing species adapted to the flowing conditions (rheophilic taxa) to non-flowing water. The importance of the disconnected pools as refuges during the dry condition was recognised to protect some endemic species and contribute to the recolonisation after the rewetting events. Include these important aquatic ecosystems in management and conservancy policies is a challenge that will contribute to preserving the freshwater resources and the biological diversity for our future generations.

**Abstract:**

Intermittent rivers and ephemeral streams (IRES) are increasingly studied because of their often-unique aquatic and terrestrial biodiversity, biogeochemical processes and associated ecosystem services. This study is the first to examine the hydrological, physicochemical and taxonomic variability during the dry-wet transition of an intermittent river in the Chilean Mediterranean Zone. Based on 30-years of river monitoring data and the TREHS tool, the hydrology of the river was characterised. Overall, the river shows a significant reduction in streamflow (−0.031 m^3^/s per year) and a substantial increase of zero flow days (+3.5 days per year). During the transition of hydrological states, variations were observed in the environmental conditions and invertebrate communities. During the drying phase, abundance, richness, and diversity were highest, while species turn-over was highest during base flow conditions. The disconnected pools and the flow resumption phases were characterised by high proportions of lentic taxa and non-insects, such as the endemic species of bivalves, gastropods, and crustaceans, highlighting the relevance of disconnected pools as refuges. Future climatic change scenarios are expected to impact further the hydrology of IRES, which could result in the loss of biodiversity. Biomonitoring and conservation programmes should acknowledge these important ecosystems.

## 1. Introduction

Intermittent rivers and ephemeral streams (IRES) or non-perennial waterways are waterways that cease to flow at a particular space and time [1,2,3], hence having distinct wet and dry periods [4,5]. They are widely distributed and considered the most common fluvial ecosystems in the world [6,7]. More than 50% of the global river network are IRES [3,8,9,10], of which monitoring mainly takes place in the temperate and Mediterranean climate zones [10,11,12]. Perturbations in the flow regime reduce the availability of mesohabitats provoking a drastic loss of biodiversity [13,14]. Mosaics of lotic, lentic and terrestrial habitats (shifting habitat mosaics) in IRES provide a complex and dynamic system, variable in time and space [15]. In analogy with the temporary pond system, this hydrological variability may alter the composition and structure of the aquatic communities and their genetic structures [16]. During flow cessation and dry period, IRES accumulate a diversity of substrates, especially of terrestrial plant litter, biofilms, animal carcases, and sediments, considered as biogeochemical reactors or hot spot areas. These substrates generate high O_2_ consumption and CO_2_ release rates, with a notable impact on the global CO_2_ flux through atmospheric emissions [17,18,19,20,21].

Mineralisation processes also result in the discharge of nutrients and suspended matter toward the coastal zone once the river gets reconnected [17,19]. In analogy with temporary pond systems, the dry riverbeds and hyporheic zone also store drought-resistant propagules, immature instars and adult stages of aquatic fauna surviving the dry conditions and recolonising the river when floods resume [22,23]. Despite some initiatives mainly in Europe, the USA and Australia that recognise the IRES, in many parts of the world, these aquatic systems remain understudied and not recognised as priority targets for conservation [6]. In the case of Chile, with an arid to semi-arid climate, there is limited knowledge of the aquatic ecology and the structure and functioning of rivers in general, despite the high demand for water in this area [24]. Although the Chilean Mediterranean Zone (ChMZ) experiences serious aridification, the increasing human population, intensification of agricultural irrigation practices, livestock farming, industrial activities and exotic forest plantations (pine and eucalyptus) demand huge freshwater volumes [24,25]. Currently, the National Water Agency (DGA, according to its initials in Spanish) is responsible for the management and administration of water resources carrying out the hydro-meteorological and water quality stations monitoring in the country. Systematic monitoring for the ecological quality is only persistent for six aquatic ecosystems (four rivers and two lakes) that count with surveillance plans associated with the secondary environmental quality standards [26]. However, more effort is necessary for monitoring the perennials or even the intermittent rivers, mainly in the ChMZ, that are very scarcely taken into account for their monitoring.

According to climate predictions, the central zone of Chile will experience an increase in summer mean air temperature from 2 to 4 °C, with a 20% to 40% reduction of precipitation and a 20% decrease in summer river streamflow [26,27,28,29,30]. The predicted hydrological changes under projected climate change and anthropogenic interventions (e.g., water abstraction) are also expected to induce perennial changes to intermittent rivers with possible repercussions for the aquatic biodiversity associated ecosystem functions [1,31,32,33]. Conventional hydro-ecological tools such as the “Indicators of Hydrologic Alteration- IHA” [34] contribute to assessing flow predictability, but currently with weak applicability in IRES. The main reason is the absence of specific indicators for the three main flow phases (flow, disconnected pools and dry riverbed).

The novel TREHS Tool (Temporary Rivers Ecological and Hydrological Status) was specially developed to characterise the various aquatic states, investigate and manage temporary rivers and assess their ecological status [31]. It uses records of at least ten years of average monthly flow series from gauging stations to characterise the type of intermittent river: *Permanent or perennial* (P), *Intermittent-pools* (I-P), *Intermittent-dry* (I-D), *Ephemeral* (E), and the six aquatic states: *Hyperrheic* (overbank flood and drift of bedload and fauna), *Eurheic* (abundant riffles and mesohabitat available and connected), *Oligorheic* (slightly connected pools with lentic and lotic fauna), *Arheic* (disconnected pools and only lentic fauna), *Hyporheic* (no surface water and terrestrial fauna active), and *Edaphic* (alluvium moisture, terrestrial fauna and resistant stages of aquatic fauna) [7,8,13,31,35,36]. 

This study aims to assess the variability of hydrological, environmental and biotic conditions in response to flow intermittence in a river from the Chilean Mediterranean zone. We based our approach on hydrological analysis and application of the TREHS tool to establish the aquatic states along the hydrological intermittence gradient and the relationship with physicochemical variables and taxonomic metrics in IRES. Furthermore, we will test to what extent the aquatic invertebrate communities use the disconnected pools as a refuge during the dry conditions and the ability to recolonise the different mesohabitats when flooding resumes. It is hypothesised that (1) the transition from flow recession to resumption will coincide with essential differences in environmental and taxonomical characteristics between the flow phases, and (2) the disconnected pools will serve as a refuge to a group of adapted taxa to the low or no flow conditions during the dry phase of the intermittent river.

## 2. Materials and Methods

### 2.1. Study Area

**Figure 1:**
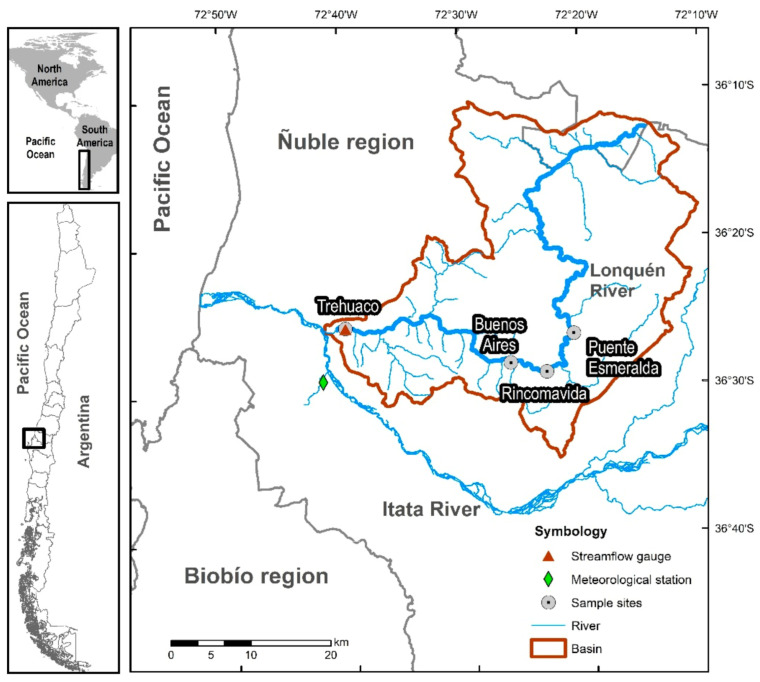
Map of the study area, the basin of the Lonquén River in Mediterranean Chile.

The Lonquén river is located in the central-south of Chile and is part of the Itata river basin (Figure 1). The drainage area is about 1178 km^2^, and the river has a Strahler stream order four [37]. According to hydrological records, the annual mean precipitation is intensive and irregular in winter with an average of 835 mm per year (lowest: 400 mm; highest: 1200 mm), mainly concentrated from June to September, with some sporadic additional rains in autumn and spring. The streamflow has a mean daily flow of 12.8 m^3^/s, ceasing from days to weeks with complete drought in summer and autumn. The mean annual air temperature is 15 °C (1 to 35 °C). December to February is the warmest period with the highest potential evapotranspiration rates, varying from 944 to 1244 mm and exceeding the average precipitation in the zone [38].

The Lonquén river originates from the coastal mountain range and is part of the interior dryland “secano interior” composed of granitic rocks from the Carboniferous period. The soils are predominantly shrink-swell clay soils, mostly degraded due to intense agricultural practices during the last centuries [39]. The land use in the basin is characterised by a cover of 20% forest (including native species and introduced plantations of pine and eucalyptus), 43% agriculture (vineyards, wheat, maise, potatoes and other vegetables), 36% meadows and thickets, and less than 1% is represented by urban, industrial and other areas without vegetation [38,40]. Rolling hills, mountains and temporary streams characterise the topography. At the same time, the geomorphology is constituted by granitic soils with low permeability. High capacity of moisture retention and low infiltration rates in the subsoil display a high superficial runoff in winter months and no runoff in summer, which rises and falls quickly after precipitation events [38]. 

### 2.2. Invertebrate Sampling

Aquatic invertebrates were sampled during the dry season (December 2015 to May 2016) and the wet period (July to October 2016). Three replicate samples of aquatic invertebrates were collected on two occasions [41] at four sites along the Lonquén River (Puente Esmeralda, Rincomavida, Buenos Aires and Trehuaco) (Figure 1 and Table 1). We considered the presence of disconnected pools during the dry period, the river connectivity and the four different aquatic states identified with the TREHS Tool to evaluate the transition from drying to flowing condition or vice versa. The 96 samples were collected using a hand net with rectangular frame (240 × 160 mm^2^; 250 µm of mesh size) following the semi-quantitative, multihabitat 3-min kick sampling method [42,43,44,45] and preserved in 70% *v/v* ethanol solution. In the laboratory, samples were sorted, and the taxonomic identification of specimens was performed to family level using standard systematic identification keys [46,47,48] and counted using a binocular dissecting stereomicroscope (Olympus SZX12). The majority of invertebrates were identified to the family level, except for Hydracarina, Copepoda, and Collembola (order level), because of the identification difficulty.

Water samples were collected at the same time as the invertebrate samples. The pH, temperature, conductivity and dissolved oxygen were recorded in situ using a multi-probe HydroLab Quanta (Hydrolab Corporation^®^, Austin, TX, USA). Suspended solids, dissolved solids, nitrate, nitrite, ammonia, total nitrogen, phosphate, and total phosphorus were analysed in the Environmental Sciences Centre EULA-Chile laboratory. Nitrite, ammonia and orthophosphate were excluded from the analysis because values were below detection limits.

### 2.3. Statistical Analysis

Daily values of thirty years (1986–2015) of precipitation and streamflow records were obtained from the meteorological station *Coelemu* (0814002-K) and the gauge station *Río Lonquén en Trehuaco* (08144001-8), both available from the Dirección General de Aguas (Ministry of Public Works in Chile: https://dga.mop.gob.cl/servicioshidrometeorologicos/Paginas/default.aspx, accessed on 1 march 2016). The software “Indicators of Hydrologic Alteration- IHA” [34] was used to estimate ecologically relevant hydrological indexes such as mean and median flow, flow predictability, mean annual minimum and maximum n-day flow (MAM-n days), and the Environmental Flow Components (EFC). These indicators were determined with non-parametric (percentile) statistics. The high flow and low flow pulse thresholds and the median plus or minus 25% were also calculated with the IHA software. Additionally, the maximum flow data series of mean daily streamflow [49] was used to predict the flood quantiles according to the Log Pearson Type III method. Finally, significant trends in streamflow and the number of zero flow days per year (threshold zero m^3^/s) were detected using the non-parametric Mann-Kendall (MK) test [50,51] and Sen’s slope estimation [52]. A confidence level of 90% was used as a threshold to categorise a positive Z value, indicating an upward trend or negative Z value showing a downward trend [53,54,55]. MK test were implemented using R software (v2.6.2) [56] and Kendall package (v2.2) [57].

We used the open-access software for PC TREHS Tool (Temporary Rivers Ecological and Hydrological Status) developed by Gallart et al. [7,31,35] to identify the temporal pattern of aquatic states related to flow. The streamflow data of the Lonquén River were analysed with the software to deduce the type of intermittent river using two metrics (flow permanence and the predictability of periods without flow). Furthermore, we obtained an estimate of each aquatic state’s percentage per month, the Temporary Regime Plot and the Aquatic States Frequency Graph. These results contributed to decide on the best period for biological monitoring and to detect variability in the invertebrate communities during drying and rewetting periods. 

The environmental variables and the community composition (presence/absence) and structure (taxonomical abundance) of the aquatic invertebrates were compared among the aquatic states identified with the TREHS model. We estimated the local diversity of the aquatic invertebrates for each aquatic state using the following measures of alpha diversity: Shannon diversity index, taxonomic richness (at the family level) and abundance. Differences among aquatic states in richness and total abundance were confirmed by the one-way ANOVA F-statistic [44]. Moreover, we calculated the proportion of the main taxonomic groups for each aquatic state, based on the taxonomical categories: Annelida, Mollusca (bivalves and gastropods), Crustacea, EPT (Ephemeroptera, Plecoptera and Trichoptera), OCH (Odonata, Coleoptera and Hemiptera), Diptera, and others to identify the groups that were most sensitive to flow permanence [15,58]. Furthermore, we calculated the dissimilarities of community composition among aquatic states employing the analysis of beta diversity (turn-over and nestedness) based on Sørensen dissimilarity to examine the species replacement and species additions for each aquatic state [15,59,60]. 

Differences between aquatic states were examined using the one-way analysis of variance (ANOVA) F-statistic. In contrast, temporal differences between individual aquatic states were conducted using Tukey’s post-hoc multiple comparisons test to identify significant differences [44]. We also compared the multivariate dispersions within the aquatic states for biological and environmental terms using the test of homogeneity of dispersion (PERMDISP; [61]) and recognise statistical differences in the mean environmental and biological distances of observations to their group centroids. We tested the null hypothesis of no differences in environmental heterogeneity, community structure (taxonomical abundance) and community composition (presence/absence) among the aquatic states. To reduce the differences between the variables, abundance, and rare taxa; this analysis was based on the Euclidean distance matrix after standardisation of data (mean = 0, SD = 1). To the environmental heterogeneity, the Bray-Curtis dissimilarity matrix used the abundance data to the community structure and the Sørensen dissimilarity matrix for the presence/absence data to the community composition [62,63,64].

Finally, a redundancy analysis (RDA) allowed us to examine the community structure and association with the distinguished environmental conditions associated with aquatic states. This constrained ordination method was based on the Bray-Curtis distance matrix after the Hellinger transformation of the abundance data [63,64,65]. RDA analysis generates a set of environmental variables (final model) explaining the community structure variation among aquatic states. The final model was selected through a forward selection procedure. This method considered two stopping rules: critical *p*-value (α = 0.05) and the value of reduced model adjusted R^2^ [66]. The Shannon diversity index, PERMDISP and RDA analysis were performed using the *vegan* package [67], and beta diversity was calculated with the *betapart* package [68] using the R software [56].

## 3. Results

### 3.1. Hydrological Variability

**Figure 2:**
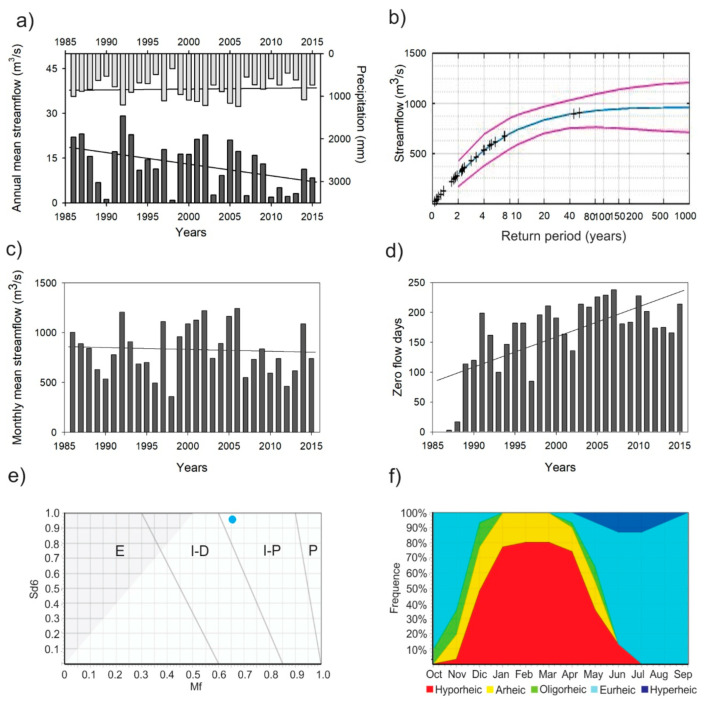
Annual mean streamflow and precipitation (**a**); probability of the extreme flood events (flood frequency analysis) showing 90% confidence limits (red line) and mean (blue line) (**b**); variation of the monthly mean streamflow (**c**); zero flow days (**d**); type of intermittent river (P: Perennial; I-P: Intermittent with pools; I-D: Intermittent-dry; E: Ephemeral) (**e**), and the aquatic states frequency graph (**f**) of the Lonquén River.

The precipitation variability shows that rainfall is concentrated between June to September with an annual mean of 831 mm (SD ± 248 mm). In 1998 the lowest rainfall was recorded (357.2 mm) and the highest in 2006 (1242.5 mm). The streamflow variability shows an annual mean daily of 12.8 m^3^/s (SD ± 8.0 m^3^/s) with the lowest values of 0.8 m^3^/s in 1998 and the highest of 29.1 m^3^/s registered to 1992. Moreover, Figure 2a) shows a substantial reduction in the annual mean daily flow. In the cases of the more significant flows, the highest streamflow in the time series data was registered at 901 m^3^/s (12 July 1987) and 909 m^3^/s (29 July 1988). Figure 2b displays the flood frequency illustrating the probability of extreme flood events in the river with a return period of 1000 years. Peak floods exceeding 900 m^3^/s are expected to take place about every 50 years in the studied river. 

The IHA analysis was run twice: a) with the flow time-series including the 176 days of zero flow and b) replacing the zero values with the no-data symbol. The first simulation showed the highest median flow values (17.20 m^3^/s) in August and zero flow (0.0 m^3^/s) from December to May. The IHA simulation, in which the zero flow values were excluded prior to the analysis, showed the highest median flow values in August (16.7 m^3^/s) and the lowest flow in January (0.1 m^3^/s). The n-day minimum range from one to 90 days of the observations was between 0.04 to 0.13 m^3^/s, respectively. In contrast, n-day maximum ranges (one to 90 days) were from 304.0 to 48.6 m^3^/s. The base flow index was estimated at 0.005 m^3^/s when zero flow values were excluded. Hence, the Environmental Flow Components (EFC) analysis revealed values of high flow peak of 19.15 m^3^/s, small flood peak of 444.5 m^3^/s and large flood peak of 901 m^3^/s (Table S1).

The MK test shows a negative trend in the monthly mean streamflow at 95% confidence level (Z = −2.08; S = −4641; *p* = 0.037) equivalent to 0.031 m^3^/s per year (Figure 2a). Instead, the zero flow days showed a positive trend at 90% confidence level (Z = 1.74; S = 148; *p* = 0.08), increasing with 3.5 days per year. The minimum of zero flow days was in 1986 (0 days) and the maximum (238 days) in 2007 with almost eight months of prolonged dry conditions (Figure 2d). These results display the considerable increased intermittence in the streamflow of the Lonquén River during the recorded time series. Additionally, the precipitation record shows no trend.

The application of the TREHS Tool revealed a Regime I-P (intermittent with pools) for the Lonquén River, as determined by the two calculated metrics: the six months seasonal predictability of dry period (Sd_6_ = 0.9593), and the measure of the flow permanence (Mf = 0.6559), as presented in the Figure 2e. The Aquatic State Frequency Graphs (Figure 2f) illustrate the flow distribution related to the aquatic state at the Lonquén River. A dry period with no surface water determined from December to April (up 78% to 100%), where the river was under Arheic and Hyporheic conditions with the presence of some isolated pools (Table S2). The wet period occurred from June to November, with predictability higher than 60% (Eurheic and Hyperheic). The low flow situation where pools are connected by a small discharge of water (Oligorheic condition) was present in April and May (rewetting or flow resumption) and from October to December (drying or flow recession). 

The distinct aquatic states were used to determine temporal variability (water quality and aquatic invertebrate composition and structure) during the transition from recession to the resumption of flow. The aquatic states were categorised according to flow permanence: Oligorheic during the drying phase or flow recession (ORD), Arheic with disconnected pools (ARH), Oligorheic during rewetting or flow resumption (ORR) and Eurheic with base flow conditions (EUR).

### 3.2. Environmental Heterogeneity 

**Figure 3:**
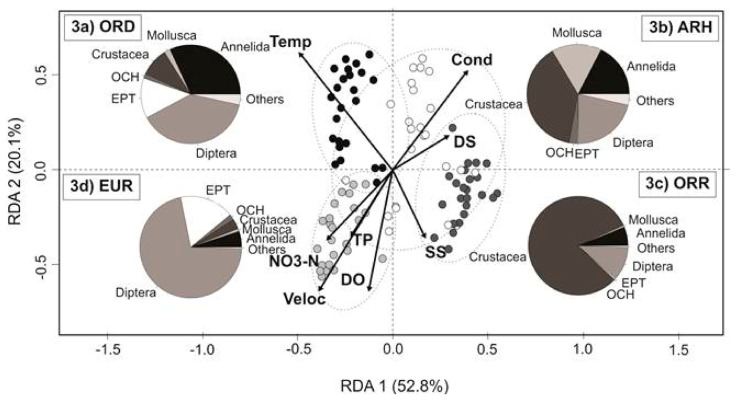
Redundancy analysis (RDA) of the environmental variables: water temperature (Temp), conductivity (Cond), dissolved solids (DS), suspended solids (SS), dissolved oxygen (DO), water velocity (Veloc), nitrate-nitrogen (NO3-N) and total phosphorus (TP). The invertebrate community for the distinguished aquatic states is represented by the composition of the major invertebrate groups (pie charts) in the Lonquén River: (**a**) ORD (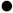 Oligorheic-drying); (**b**) ARH (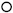 Arheic); (**c**) ORR (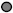 Oligorheic-rewetting), and (**d**) EUR (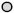 Eurheic).

Differences among environmental variables and aquatic states are summarised in Figure 3 and Table 2. The first two axes of the redundancy analysis (RDA1 and RDA2) significantly explained 72.9% of the total variation of environmental and taxonomical components (*p* < 0.001). RDA1 (52.8%) was mostly associated with velocity and temperature, the two variables that contributed most to environmental variability concomitant to the EUR and ORD, respectively. Conductivity and dissolved solids were the essential variables that explained the variability of the ARH states and were loaded toward the positive dimension of RDA1 and RDA2. The ORR state represented a transition between the dry and wet period with high values of dissolved oxygen and suspended solids, while the EUR state was also characterised by high values of dissolved oxygen, nitrates, total nitrogen and total phosphorus.

### 3.3. Taxonomical Heterogeneity 

**Figure 4:**
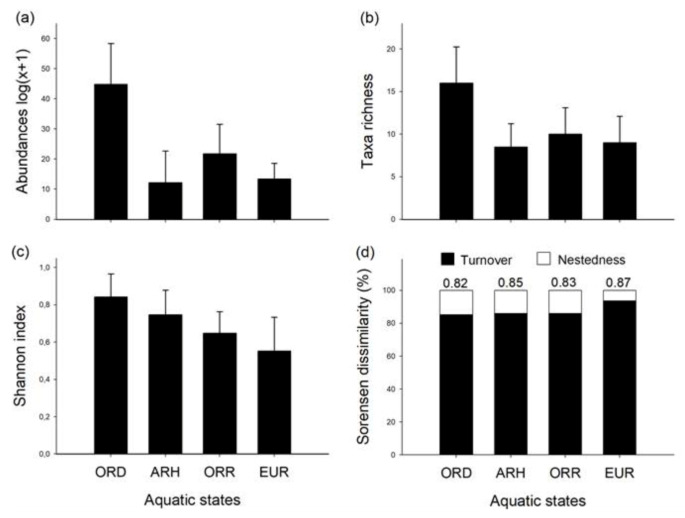
Mean of taxonomic metrics with standard deviation (—) for the distinguished aquatic states in the Lonquén River. Abundance log (x + 1) (**a**); taxa richness (**b**); Shannon index (**c**); Sorensen dissimilarity (Turn-over %) (**d**) (see acronyms of aquatic states in Figure 3).

A total of 66,392 individuals belonging to 49 families were collected in 96 samples. With respect to the aquatic states, the majority of individuals was obtained in the ORD state (absolute; relative abundance, 51,503; 77.6%), followed by the ORR (10,440; 15.7%), while the EUR (2326; 3.5%) and ARH states (2123; 3.2%) hosted the lowest numbers of individuals. These differences were statistically significant between the aquatic states (one-way ANOVA; F = 22.59, *p* < 0.001) with a significant increase marked to the ORD in comparison to the ARH (Tukey’s post-hoc test; F = 7.5, *p* < 0.001), ORR (Tukey’s post-hoc test; F = −5.8, *p* < 0.001) and EUR (Tukey’s post-hoc test; F = 6.5, *p* < 0.001) (Figure 4a). Similarly, family richness was most distinct during ORD in comparison to the rest of the aquatic states (one-way ANOVA; F = 8.995, *p* < 0.001), showing highest mean values (mean ± SD; 15.7 ± 4.5; total = 40) in contrast with the ORR (10.0 ± 3.2; total = 32) (Tukey´s post-hoc test; F = −1711, *p* < 0.005), EUR (9.2 ± 2.8; total = 29) (Tukey´s post-hoc test; F = 2049, *p* < 0.001) and ARH states (8.2 ± 3.0; total = 28) (Tukey´s post-hoc test; F = 2058, *p* < 0.001) (Figure 4b). The Shannon index (Figure 4c) revealed highest diversity in the drying condition (ORD), with decreasing values in the disconnected pools (ARH), rewetting phase (ORR) and base flow (EUR). The latter state was characterised by the highest beta diversity (Sørensen), with high percentages of species turn-over as compared to the other aquatic states (Figure 4d).

The aquatic invertebrate community was most distinguished in the ORD, with a high proportion of Annelida, crustaceans (cladocerans, copepods and ostracods), EPT (mainly mayflies) and Diptera (chironomids), superior to the rest of the aquatic states (Figure 3a). In the ARH state, the crustaceans, annelids, dipterans and molluscs were dominant in the stagnant pools (Figure 3b), as well as in the ORR, where microcrustaceans showed a higher abundance of individual species (Figure 3c). The EUR state was distinguished by the abundance of Diptera (chironomids) and Ephemeroptera (mayflies) with a reduced presence of Mollusca (gastropods), Ostracoda, Plecoptera (Grypopterigidae), Coleoptera (Dytiscidae) and non-chironomid dipterans (Figure 3d).

**Figure 5:**
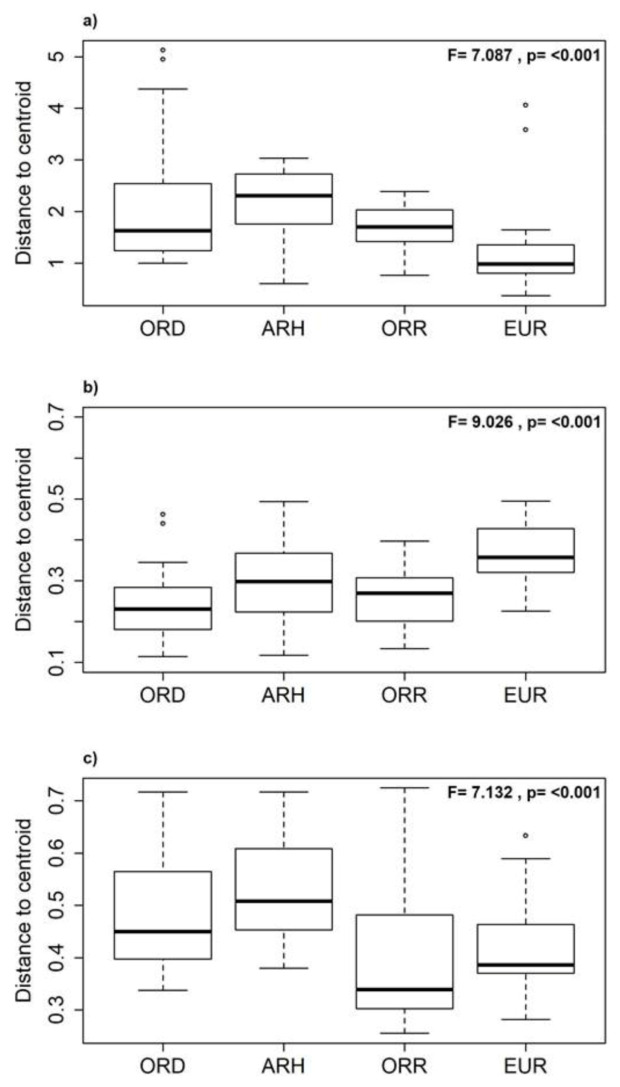
Boxplots of the test of homogeneity (PERMDISP) representing the mean distance from groups centroids and the outliers values (◦) of the environmental heterogeneity with Euclidean distance (**a**); taxonomical structure using abundance and Bray-Curtis dissimilarity (**b**), and taxonomical community using presence/absence and Sørensen dissimilarity (**c**) of the aquatic states (see acronyms of aquatic states in Figure 3).

A high proportion of EPT and insect taxa were observed in the ORD and EUR states compared to the OCH and non-insect taxa (Figure 3a,d). The latter were mostly represented in the disconnected pools and during flow resumption (Figure 3b,c). The test of homogeneity (PERMDISP) evidenced the significant statistical differences in environmental heterogeneity among aquatic states (F = 7.087; *p* < 0.001; Figure 5a). The same-pattern was revealed for the community composition (presence/absence; F = 9.026; *p* < 0.001; Figure 5b) and the community structure (Bray-Curtis; F = 7.132; *p* < 0.001; Figure 5c) among the aquatic states.

## 4. Discussion

Here we present for the first time the integration of aquatic diversity and environmental conditions associated with different hydrological states—from drying to flow resumption—in an intermittent river from the Chilean Mediterranean. Significant patterns were revealed that are confirmed by several studies in other Mediterranean-climate regions of the world [10,14,69,70,71,72,73]. Besides, the open-access software TREHS Tool displayed an excellent response to the flow regime temporality in the Lonquén River compared to other hydrological indexes (e.g., Indicators of Hydrological Alterations—IHA). The tool included qualitative features such as the presence of flow, isolated pools or the lack of surface water, relevant to the biological communities [7]. Even though the Lonquén River is not a Mediterranean intermittent river from the Northern Hemisphere, the tool also proved to be applicable in our study region, as it reliably generated the two metrics and the different aquatic states. Further comparisons with other Mediterranean intermittent rivers could be considered, such as the Celone River (Puglia, Italy), which has similar seasonal predictability and flow permanence as compared to the Lonquén River (Figure 2e,f), for a better understanding on the functioning and structure of Mediterranean rivers in general [7,31,74]. 

Acuña et al. [75] and Buffagni et al. [76] observed a maximum habitat heterogeneity or diversification during streamflow reduction with the drying of the intermittent river. Drying of the river induced changes in the invertebrate community structure and composition with dominance of tolerant and adapted taxa to the low or no flow conditions and with the capacity to resist and recover from the drought period [72,76,77,78,79]. Our results confirmed this general pattern (Figure 3a). During the drying or recession period (ORD), there was a dominance of Diptera (chironomids), Annelida (*Nais* sp.) and Ephemeroptera (Baetidae and Caenidae) with a smaller but still considerable proportion of Ostracoda (Cyprididae), Hydracarina, Copepoda (Harpacticoida), Branchiopoda (*Daphnia ambigua*), Gastropoda (*Physa chilensis*), Hemiptera (Corixidae and Belastomatidae), Odonata (Aeshnidae and Coenagrionidae) and Coleoptera (Dytiscidae). All of these groups are recognised to be more adapted to the cessation of streamflow and to be able to survive lentic conditions [60,69]. The Ephemeroptera Baetidae family is also present during drying conditions and can resist prolonged harsh conditions while gaining tolerance to drought [80]. In our study, the Baetidae family (8%) and Caenidae (5%) also showed good representation during flow recession in the Lonquén River. 

Prior studies conducted by Munné & Prat in 184 sites located in the Catalan Mediterranean Basins, NE Spain [70] and Hill & Milner in Manifold and Hamps rivers in the English Peak District of United Kingdom [60] reported that during the transition from the lotic to the lentic phase (disconnected, isolated or stagnant pools), many rheophilic taxa are replaced by others that are adapted to lentic habitats and that actively disperse when the river falls dry, such as Hemiptera (Corixidae) and Coleoptera (Dytiscidae and Helephoridae). A similar pattern was observed in our results, specifically illustrated by the higher presence of Hemiptera (Corixidae and Belastomatidae), Coleoptera (Heteroceridae, Hydrophilidae, Dytiscidae and Helephoridae) and Odonata (Coenagrionidae, Aeshnidae and Libelullidae) in comparison with the Ephemeroptera, Plecoptera and Trichoptera. 

During drying and rewetting events, complex interactions take place between hydrological and physicochemical processes that expose the aquatic organisms to changing levels of abiotic variables, including chemical compounds [81]. Our results showed significant differences with a higher temperature during the drying period (ORD) (mean ± SD; 27.9 ± 3.2 °C) as part of the seasonal variability (summer). During the transition of drying to isolated pools (ARH), lower values of dissolved oxygen (5.95 ± 2.74 mg/L), suspended solids (3.89 ± 1.62 mg/L) and high conductivity values (289 ± 28 µS/cm) were observed in the pools (Table 2). These changes associated with the loss of flow permanence also cause distinct variability in photosynthetic and respiration processes that may, in turn, result in high values of dissolved oxygen and pH on some occasions during the day and lower values at night [18,70]. These sometimes-extreme environmental conditions affect the richness, abundance and diversity of aquatic invertebrates [76], as revealed here by the significant changes that were observed between the recession of flow to the disconnected pools phase (accept hypothesis 1; Figure 3 and Figure 4).

When flow ceases in IRES, the presence of disconnected pools serves as an essential refuge to escape drought for a diverse and unique aquatic flora and fauna [18,82], which are adapted to survive even in the dry streambed through diverse strategies [60]. As a consequence, the density of lentic taxa increases, also including predator species, such as odonate larvae (Aeshnidae) and water scavenger beetles (dytiscids) [83]. Nevertheless, some fishes were present in the isolated pools of our study, which are also recognised to influence as predators and generate a significant change in the density and assemblages of the aquatic invertebrates [83]. 

Rest-pools in the streambed also provide habitat for newly colonising and dispersing taxa with the opportunity to survive and persist through flow cessation [18,60,82,84]. Although in our findings, some species of Diptera, Annelida and Ephemeroptera occurred in higher proportions during the drying period (Figure 3b). Peculiarly, high abundances were present in pools of taxa belonging to Gastropoda (*Physa chilensis*), Annelida (Lumbriculidae and Naiididae), Ostracoda (Cyprididae), Copepoda (Cyclopoida and Harpacticoida) and Branchiopoda (Daphniidae) with fewer individuals counted for Coleoptera (Dytiscidae and Hydrophilidae) and Odonata (Aeshnidae). Decapoda (*Aegla* sp. and *Samastacus spinifrons*) and Bivalvia (*Diplodon chilensis*) were only represented in the isolated pools during the dry phase, where they have a low dispersal capacity and are protected from flow recession (accept hypothesis 2). 

The flow resumption phase in intermittent rivers allows the recolonisation after the drought event. These routes have been distinguished by aerial dispersal, drift from upstream, redistribution from instream refugia or diapause (hyporheic zone) with increases in abundance and diversity of macroinvertebrates such as midges (chironomids) and blackflies (simulids) that are abundant in the early stage of flow resumption [62,85]. The invertebrate community structure in intermittent rivers typically recovers within a month after flow resumption, except for populations with low resilience, resulting in adverse effects on ecosystem functions (e.g., mollusc and some crustaceans) [79,86,87]. However, the resistant propagules in the sediment (often called seedbanks) of dry riverbeds and from the hyporheic zone are crucial for recolonisation after flow resumption and determine the aquatic invertebrate resilience level after drought [72,88,89,90]. In this study, a higher proportion of Branchiopoda (Bosminidae), Ostracoda (Cyprididae), Copepoda (Cyclopoida) and Annelida (Lumbriculidae) occurred during the rewetting, in contrast to the disconnected pools phase. Branchiopoda, Ostracoda and Copepoda produce drought-resistant dormant eggs or larval stages that survive long in the sediment and hatch after flow resumption. This process makes these species more resilient to drought than other taxa, which is in analogy with endorheic temporary pond systems [22]. 

During the wet season (base flow), recovery of flow permanence in the intermittent river contributes to the formation of spatial heterogeneity of mesohabitats, restoring the riffle habitats and promoting the recolonisation of riffles [71]. Rheophilic taxa (EPT) tends to be dominant in these mesohabitats, where especially a high correlation is reported between EPT and the increase of flow permanence [91]. In contrast, taxa adapted to low flow (OCH) disappear. Similar to Kelso and Entrekin [84], there is an increased richness and diversity of rheophilic taxa during rewetting. However, the richness and abundance during this (base flow) phase in our studied system were lower in comparison with the drying and rewetting conditions (Figure 4), which may be due to the disturbance by high flood in the river before the sampling dates (hyperrheic state). Such flooding events are recognised to cause intense but short-lived disturbance [31], causing a considerable scour of the aquatic invertebrates. In the Eurheic state, Leptophlebidae, Gripopterigidae, Simuliidae, Limoniidae and Tabanidae were recorded, representing riffle taxa adapted to flow [92].

Our results also demonstrated that family taxon richness and abundance metrics were reliable indicators to compare between the aquatic states and unravel variations during the transition from drying to rewetting and base flow, similarly reported by Munné & Prat [70]. Moreover, proportions of aquatic invertebrate groups (e.g., EPT%, OCH%, Diptera%, Non-insect%) and the diversity indices (alpha and beta) responded to the contraction and expansion of flow permanence in the Lonquén River (Table 3). We found higher richness, abundance and diversity during the drying period (flow recession) than during flowing conditions. These patterns will allow comparison with other IRES [71,72,93].

Some studies state that the loss of freshwater biodiversity at a global scale is expected to be higher in Mediterranean basins, especially considering climate change and associated anthropogenic threats, with habitat loss as the prime factor resulting in species extinction [33,71,97]. The predictions of climate change for central Chile include extreme climatic events, such as the increase in summer mean air temperature (2 to 4 °C), reduction of precipitation (20 to 40%) and decrease in summer river streamflow (20%) [26,27,28,29,30], especially in the Mediterranean zone [28,29]. Already in our study, a reduction of streamflow and an increase in the number of zero flow days were revealed in the Lonquén River during a period of about 30 years. It is expected that this pattern will continue, further affecting the flow permanence in the region under future climatic change and land-use scenarios. These predicted harsh conditions are expected to decrease the percentages of Eurheic and Oligorheic states, while at the same time to increase the prevalence of Arheic and Hyporheic (dry period) conditions.

Future reductions in water permanence may lead to increased habitat heterogeneity with contraction of flow and formation of pools with the need of migration and prolonged exposure of aquatic organisms to predation and competition in isolated pools or even the disappearance of these refugees by completely drying-out [87,98,99,100,101]. Some resistant species could become more dominant in the dry riverbeds. For example, zooplankton resists dry periods by producing resistant dormant eggs that can be distributed by birds and winds and that hatch when floods return [16]. Indeed, branchiopods and ostracods were observed in higher proportions in the Oligorheic state after flow resumption (ORR) in our results, indicating the importance of resistance traits to the dry conditions, which may even become more intense with possible scenarios of climate change [102]. Furthermore, the extent and duration of drying events and water abstraction will further lower the water table and affect the colonisation and resilience of the invertebrate communities in IRES from the hyporheic zone that is also used as a refuge and to survive during the drying events [88]. 

Although the central Chile Mediterranean basin (ChMZ) is recognised as a global hotspot of biodiversity with extraordinary endemism in plants and fauna [97,103], only limited research is taking place on IRES, essential ecosystems in the region. Studies on freshwater biodiversity are scarce, and numerous Mediterranean rivers in Chile remain completely unexplored or not including an ecological approach [24]. Unfortunately, as for most of the worldwide Mediterranean systems, the IRES have not yet been included in conservation management despite the ecosystem services that provide for the human-well being [104], where the standards for water quality and biodiversity conservation are only determined for perennial rivers [71], especially concerning their vulnerability to climate change and anthropogenic stressors [105].

However, limited knowledge, availability of resources for research and development in most Mediterranean countries have generated ineffective conservation and management strategies of IRES [10]. It is necessary that public and private institutions relevant to water monitoring and management in Chile, such as the DGA, can recognise the ecosystem services provided by IRES and extend the ecological monitoring to these streams. Establishing reference conditions of IRES in the ChMZ will be an essential asset for conservation and management in response to the climate change, considering the decline of endemic populations, the loss of freshwater biodiversity and fragmentation of the aquatic ecosystems. Some of the bivalves, gastropods and crustacean species in our study are endemics for Chile, should be considered in adaptative and conservation strategies for their low capability to migrate to other basins under harsh climate conditions [24,106]. These considerations are especially important for the species *Diplodons chilensis, Littoridina cumingii, Chilina dombeyana, Physa chilensis, Biomphalaria chilensis, Gundlachia gayana, Hyalella costera, Aegla sp.* and *Samastacus spinifrons*, additionally to the microcrustacean groups (cladocerans, copepods and ostracods) identified in our samples (Table S3). This is especially relevant, considering the expectation that more perennial rivers will convert to temporal or intermittent ones by the impact of land use and climate change in the region [32].

## 5. Conclusions

This study is the first on IRES integrating hydrological, environmental and aquatic diversity variables during the transition from drying to flowing condition in a Mediterranean intermittent river in South America. Significant patterns revealed that help to understand the response of aquatic invertebrates to shifting temporal habitats. Our findings revealed that a substantial reduction of the streamflow and a clear trend towards increasing zero flow days in the last decades in the Mediterranean climates could cause lost sensible species with low capability to migrate on the riverine ecosystems. Furthermore, it is expected to find alterations in biological diversity and their biotic interactions. It was also possible to affirm the importance of disconnected pools as refuges for a diverse and unique set of aquatic flora and fauna and provide habitat for colonisation after flow resumption. During the transition from recession to the resumption of flow, the biological diversity significantly changed from species adapted to flowing conditions (rheophilic taxa) to non-flowing water such as the OCH taxa and the non-insect species (bivalves, gastropods and crustacean). Further long-term biological, hydro-meteorological and water quality monitoring studies are necessary to provide a site-specific history that allows the integration of temporal and spatial variability to define the conservation status and identify climatic change effects.

## Figures and Tables

**Table 1:** Location of the sampling sites of the Lonquén River and distance to source and downstream confluence.

Sampling Sites/Meteorological and Gauge Stations	Geographic Coordinates		Altitude (masl)	Max Wet River Wide (m)	Distance to:	
	Latitude	Longitude			Source (km)	Downstream Confluence (km)
Puente Esmeralda	−36.442051	−72.351664	80	40	45.0	44.8
Rincomavida	−36.480823	−72.383816	65	40	52.9	36.9
Buenos Aires	−36.471718	−72.441632	60	70	59.6	30.2
Trehuaco	−36.427605	−72.663469	30	100	86.3	3.5
Coelemu	−36.491944	−72.698888	80			
Río Lonquén en Trehuaco	−36.427665	−72.664110	30			

**Table 2:** Summary of the environmental variables (mean ± standard deviation) measured in the different aquatic states at Table 2015. (see acronyms of aquatic states in Figure 3).

	ORD	ARH	ORR	EUR
Current velocity (m/s)	0.013 ± 0.03	0.0 ± 0.0	0.093 ± 0.08	0.516 ± 0.10
pH	7.64 ± 0.40	7.52 ± 0.68	6.65 ± 0.24	7.80 ± 0.27
Temperature (°C)	27.9 ± 3.2	15.7 ± 1.6	10.4 ± 1.5	14.2 ± 0.1
Conductivity (µS/cm)	232 ± 48	289 ± 28	226 ± 38	120 ± 4.5
Dissolved oxygen (mg/L)	5.48 ± 1.53	5.96 ± 2.74	8.72 ± 2.05	10.81 ± 0.23
Suspended solids (mg/L)	9.76 ± 5.19	3.89 ± 1.62	21.4 ± 9.31	15.14 ± 2.83
Dissolved solids (mg/L)	164 ± 63	184 ± 21	172 ± 17	127 ± 7
Nitrate-NO_3_-N (mg/L)	0.06 ± 0.10	0.12 ± 0.07	0.02 ± 0.01	0.17 ± 0.02
Total Nitrogen (mg/L)	0.40 ± 0.14	0.27 ± 0.20	0.26 ± 0.10	0.53 ± 0.16
Total Phosphate (mg/L)	0.05 ± 0.01	0.10 ± 0.07	0.06 ± 0.04	0.15 ± 0.06

**Table 3:** Taxonomic metrics selected by relevance to intermittent flows and expected response to the transitional variability from flow recession to the resumption in IRES.

Metric	Definition	Relevance to Intermittent Flows	Reference
Richness	The number of species of a given taxon in the chosen assemblage. The number of species or taxa in the unit of study [94].	Lower values are expected in intermittent flow rivers than in permanent ones.	[72,93]
		It is decreasing after the disconnection of the river in isolated pools with lentic-like and resistance taxa colonising in the dry period.	[10,14,60,95]
Abundance	Number of individuals (density or biomass) of each specie or community [94].	Lower values are expected in intermittent flow rivers than in permanent ones.	[62,71,72]
			[75]
		Increased abundance (and richness) is possible to find soon after flow ceased with a rapid decrease when the isolated pools are constituted.	Present study
Shannon diversity	Mathematical index to measure the diversity in a natural systems and it assumes that individuals are randomly sampled from an infinitely large community and that all species are represented in the sample [94].	Higher values are expected in perennial sites than intermittent.	[93]
		It is expected to find high values in the drying condition or pools when the river is recently disconnected.	Present study
Beta diversity	Difference in species composition (and sometimes species abundance) among sites, or turn-over between two or more habitats or localities [94]. Turn-over: Replacement of some species by others between sites Nestedness: smaller numbers of species are subsets of the biota at richer sites [59].	Change in community composition along hydrological intermittence gradients is driven by loss (nestedness) and turnover (replacement) of taxa due to increasing fragmentation or environmental harshness.	[15,60,96]
		Community structure may vary sharply during the different hydrological phases. During the phases dominated by dispersal (flowing), the nestedness may be observed, particularly for weak to moderate dispersers. In contrast, when species sorting or environmental filtering dominates in IRES, the taxa turn-over may be observed more commonly during the non-flowing or dry phase.	[96]
		High beta diversity (turn-over) is expected to find in the perennial sites after the high flood perturbation. However, the nestedness is possible to be moderate in intermittent sites and disconnected pools than perennials.	Present study

## Data Availability

The data presented in this study are available on request from the corresponding author.

## Supplementary Materials

The following are available online at https://www.mdpi.com/article/10.3390/biology10040316/s1, Table S1: Indicators of Hydrologic Alteration-IHA in the Lonquén River. Table S2: Percentage of the aquatic states frequency of the Lonquén River. Table S3: Aquatic invertebrate taxa (Family and taxon) recorded in the Lonquén River, according to aquatic states and sampling sites (PE: Puente Esmeralda; RM: Rincomavida; BA: Buenos Aires; TR: Trehuaco).

## References

[1] Larned S.T., Datry T., Arscott D.B., Tockner K. (2010). Emerging concepts in temporary-river ecology. Freshw. Bio..

[2] Acuña V., Datry T., Marshall J., Barceló D., Dahm C.N., Ginebreda A., McGregor M., Sabater S., Tockner K., Palmer M.A. (2014). Why should we care about temporary waterways?. Science.

[3] Datry T., Larned S.T., Tockner K. (2014). Intermittent rivers: A challenge for freshwater ecology. BioScience.

[4] Sánchez-Montoya M., Gómez R., Suárez M., Vidal-Abarca M., Elliot H.S., Martin L.E. (2011). Ecological Assessment of Mediterranean Streams and the Special Case of Temporary Streams. River Ecosystems: Dynamics, Management and Conservation.

[5] Arthington A., Bernardo J., Ilhéu M. (2014). Temporary rivers: Linking ecohydrology, ecological quality and reconciliation ecology. River Res. Appl..

[6] Leigh C., Boulton A.J., Courtwright J.L., Fritz K., May C.L., Walker R.H., Datry T. (2016). Ecological research and management of intermittent rivers: A historical review and future directions. Freshw. Bio..

[7] Gallart F., Cid N., Latron J., Llorens P., Bonada N., Jeuffroy J., Jiménez-Argudo S.M., Vega R.M., Solà C., Soria M. (2017). TREHS: An open-access software tool for investigating and evaluating temporary river regimes as a first step for their ecological status assessment. Sci. Total Environ..

[8] Prat N., Gallart F., Von Schiller D., Polesello S., García-Roger E.M., Latron J., Rieradevall M., Llorens P., Barberá G.G., Brito D. (2014). The MIRAGE Toolbox: An integrated assessment tool for temporary streams. River Res. Appl..

[9] Datry T., Fritz K., Leigh C. (2016). Challenges, developments and perspectives in intermittent river ecology. Freshw. Bio..

[10] Skoulikidis N.T., Sabater S., Datry T., Morais M.M., Buffagni A., Dörflinger G., Zogaris S., Sánchez-Montoya M.M., Bonada N., Kalogianni E. (2017). Non-perennial Mediterranean rivers in Europe: Status, pressures, and challenges for research and management. Sci. Total Environ..

[11] Buttle J.M., Boon S., Peters D.L., Spence C., Van Meerveld H.J., Whitfield P.H. (2012). An overview of temporary stream hydrology in Canada. Can. Water Resour. J..

[12] Steward A.L., Negus P., Marshall J.C., Clifford S.E., Dent C. (2018). Assessing the ecological health of rivers when they are dry. Ecol. Indic.

[13] De Girolamo A.M., Gallart F., Pappagallo G., Santese G., Lo Porto A. (2015). An eco-hydrological assessment method for temporary rivers. The Celone and Salsola rivers case study (SE, Italy). Ann. Limnol..

[14] Soria M., Leigh C., Datry T., Bini L.M., Bonada N. (2019). Biodiversity in perennial and intermittent rivers: A meta-analysis. Oikos.

[15] Datry T., Moya N., Zubieta J., Oberdorff T. (2016). Determinants of local and regional communities in intermittent and perennial headwaters of the Bolivian Amazon. Freshw. Bio..

[16] Brendonck L., Pinceel T., Ortells R. (2017). Dormancy and dispersal as mediators of zooplankton population and community dynamics along a hydrological disturbance gradient in inland temporary pools. Hydrobiologia.

[17] Tzoraki O., Nikolaidis N.P., Amaxidis Y., Skoulikidis N.T. (2007). In-stream biogeochemical processes of a temporary river. Environ. Sci. Technol..

[18] Skoulikidis N.T., Vardakas L., Amaxidis Y., Michalopoulos P. (2017). Biogeochemical processes controlling aquatic quality during drying and rewetting events in a Mediterranean non-perennial river reach. Sci. Total Environ..

[19] Datry T., Foulquier A., Corti R., von Schiller D., Tockner K., Mendoza-Lera C., Clément J.C., Gessner M.O., Moleón M., Stubbington R. (2018). A global analysis of terrestrial plant litter dynamics in non-perennial waterways. Nat. Geosci..

[20] Shumilova O., Zak D., Datry T., von Schiller D., Corti R., Foulquier A., Obrador B., Tockner K., Allan D.C., Alternatt F. (2019). Simulating rewetting events in intermittent rivers and ephemeral streams: A global analysis of leached nutrients and organic matter. Glob. Chang. Biol..

[21] von Schiller D., Datry T., Corti R., Foulquier A., Tockner K., Marcé R., García-Baquero G., Odriozola I., Obrador B., Elosegi A. (2019). Sediment respiration pulses in intermittent rivers and ephemeral streams. Glob. Biogeochem. Cycles.

[22] Brendonck L., De Meester L. (2003). Egg banks in freshwater zooplankton: Evolutionary and ecological. Hydrobiologia.

[23] Stubbington R. (2012). The hyporheic zone as an invertebrate refuge: A review of variability in space, time, taxa and behaviour. Mar. Freshw. Res..

[24] Figueroa R., Bonada N., Guevara M., Pedreros P., Correa-Araneda F., Díaz M.E., Ruiz V.H. (2013). Freshwater biodiversity and conservation in Mediterranean climate streams of Chile. Hydrobiologia.

[25] Bonada N., Resh V.H. (2013). Mediterranean-climate streams and rivers: Geographically separated but ecologically comparable freshwater systems. Hydrobiologia.

[26] Pastén P., Vega A., Guerra P., Pizarro J., Lizama K. (2019). Water quality in Chile: Progress, challenges and perspectives. Water Quality in the Americas: Risk and Opportunities.

[27] CONAMA (2006). Estudio de la Variabilidad Climática en Chile para el Siglo XXI.

[28] Garreaud R. (2011). Cambio Climático: Bases físicas e impactos en Chile. Rev. Tierra Adentro-Inia.

[29] Valdés-Pineda R., Pizarro R., García-Chevesich P., Valdés J.B., Olivares C., Vera M., Balocchi F., Pérez F., Vallejos C., Fuentes R. (2014). Water governance in Chile: Availability, management and climate change. J. Hydrol..

[30] Cabré M.F., Solman S., Núñez M. (2016). Regional climate change scenarios over southern South America for future climate (2080–2099) using the MM5 Model. Mean, interannual variability and uncertainties. Atmósfera.

[31] Gallart F., Prat N., García-Roger E.M., Latron J., Rieradevall M., Llorens P., Barbera G.G., Brito D., de Girolamo A.M., Lo Porto A. (2012). A novel approach to analysing the regimes of temporary streams in relation to their controls on the composition and structure of aquatic biota. Hydrol. Earth Syst. Sci..

[32] Datry T., Larned S.T., Fritz K.M., Bogan M.T., Wood P.J., Meyer E.I., Santos A.N. (2014). Broad-scale patterns of invertebrate richness and community composition in temporary rivers: Effects of flow intermittence. Ecography.

[33] Cid N., Verkaik I., García-Roger E.M., Rieradevall M., Bonada N., Sánchez-Montoya M.M., Gómez R., Suárez M.L., Vidal-Abarca M.R., Demartini D. (2016). A biological tool to assess flow connectivity in reference temporary streams from the Mediterranean Basin. Sci. Total Environ..

[34] Richter B.D., Baumgartner J.V., Powell J., Braun D.P. (1996). A method for assessing hydrologic alteration within ecosystems. Conserv. Bio..

[35] Gallart F., Llorens P., Latron J., Cid N., Rieradevall M., Prat N. (2016). Validating alternative methodologies to estimate the regime of temporary rivers when flow data are unavailable. Sci. Total Environ..

[36] Tzoraki O., De Girolamo A.M., Gamvroudis C., Skoulikidis N. (2016). Assessing the Flow alteration of temporary streams under current conditions and changing climate by SWAT model. Int. J. River Basin Manag..

[37] División de Estudios y Planificación (2011). Regionalización de Parámetros en Cálculo de Escorrentía en Cuencas Pluviales.

[38] Uribe H., Pérez C., Okuda Y. (2004). Recursos Hídricos y Manejo del Agua para un Desarrollo Sustentable del Secano.

[39] Stewart R.D., Abou Najm M.R., Rupp D.E., Lane J.W., Uribe H.C., Arumí J.L., Selker J.S. (2015). Hillslope run-off thresholds with shrink-swell clay soils. Hydrol. Process..

[40] Duque L.F., Vázquez R.F. (2015). Modelización Hidrológica de una Cuenca Intermitente ubicada en la VIII Región de Chile. Rev. Politcnica.

[41] McCabe D.J. (2011). Sampling biological communities. Nat. Educ. Knowl..

[42] Reznickova P., Paril P., Zahradkova S. (2007). The ecological effect of drought on the macroinvertebrate fauna of a small intermittent stream an example from the Czech Republic. Int. Rev. Hydrobiol..

[43] Argyroudi A., Chatzinikolaou Y., Poirazidis K., Lazaridou M. (2009). Do intermittent and ephemeral Mediterranean rivers belong to the same river type?. Aquat. Ecol..

[44] Stubbington R., Wood P.J., Boulton A.J. (2009). Low flow controls on benthic and hyporheic macroinvertebrate assemblages during supra-seasonal drought. Hydrol. Process..

[45] White J.C., Hannah D.M., House A., Beatson S.J.V., Martin A., Wood P.J. (2017). Macroinvertebrate responses to flow and stream temperature variability across regulated and non-regulated rivers. Ecohydrology.

[46] Merrit R.W., Cummins K.W. (1996). An Introduction to the Aquatic Insects of North America.

[47] Domínguez E., Molineri C., Pescador M.L., Hubbard M.D., Nieto C., Adis J., Arias J.R., Rueda-Delgado G., Wantzen K.M. (2006). Ephemeroptera of South America. Aquatic Biodiversity of Latin America (ABLA).

[48] Domínguez E., Fernández H.R. (2009). Macroinvertebrados Bentónicos Sudamericanos.

[49] Parkes B., Demeritt D. (2016). Defining the hundred year flood: A Bayesian approach for using historic data to reduce uncertainty in flood frequency estimates. J. Hydrol..

[50] Mann H.B. (1945). Nonparametric tests against trend. Econometrica.

[51] Kendall M.G. (1975). Rank Correlation Methods.

[52] Sen P.K. (1968). Estimates of the regression coefficient based on Kendall’s tau. J. Am. Stat. Assoc..

[53] Kundzewicz Z.W., Graczyk D., Maurer T., Pińskwar I., Radziejewski M., Svensson C., Szwed M. (2005). Trend detection in river flow series: 1. Annual maximum flow. Hydrol. Sci. J..

[54] Bouza-Deaño R., Ternero-Rodríguez M., Fernández-Espinoza A.J. (2008). Trend study and assessment of surface water quality in the Ebro River (Spain). J. Hydrol..

[55] Shahid S. (2010). Rainfall variability and the trends of wet and dry periods in Bangladesh. Int. J. Clim..

[56] R Development Core Team (2016). R: A Language and Environment for Statistical Computing, Version 2.6.2..

[57] McLeod A.I. (2011). Kendall Rank Correlation and Mann-Kendall Trend Test. R Kendall Package Version 2.2. https://cran.r-project:web/packages/Kendall/Kendall.pdf.

[58] Álvarez-Cabria M., Barquín J., Juanes J.A. (2010). Spatial and seasonal variability of macroinvertebrate metrics: Do macroinvertebrate communities track river health?. Ecol. Indic.

[59] Baselga A. (2010). Partitioning the turn-over and nestedness components of beta diversity. Glob. Ecol. Biogeogr..

[60] Hill M.J., Milner V.S. (2018). Ponding in intermittent streams: A refuge for lotic taxa and a habitat for newly colonising taxa?. Sci. Total Environ..

[61] Anderson M.J. (2006). Distance-based tests for homogeneity of multivariate dispersions. Biometrics.

[62] Stubbington R., Greenwood A.M., Wood P.J., Armitage P.D., Gunn J., Robertson A.L. (2009). The response of perennial and temporary headwater stream invertebrate communities to hydrological extremes. Hydrobiologia.

[63] Heino J., Grönroos M., Ilmonen J., Karhu T., Niva M., Paasivirta L. (2013). Environmental heterogeneity and β diversity of stream macroinvertebrate communities at intermediate spatial scales. Freshw. Sci..

[64] Jirkänkallio-Mikkola J., Heino J., Soininen J. (2016). Beta diversity of stream diatoms at two hierarchical spatial scales: Implications and biomonitoring. Freshw. Bio..

[65] Legendre P., Gallagher E.D. (2001). Ecologically meaningful transformations for ordination of species data. Oecologia.

[66] Blanchet G., Legendre P., Borcard D. (2008). Forward selection of spatial explanatory variables. Ecology.

[67] Oksanen J., Blanchet F.G., Friendly M., Kindt R., Legendre P., McGlinn D., Minchin P.R., O’Hara R.B., Simpson G.L., Solymos P. Vegan: Community Ecology Package. R Package Version 2.4-0. http://CRAN.R-project:package=vegan.

[68] Baselga A., Orme D.L. (2012). Betapart: A R package for the study of beta diversity. Methods Ecol. Evol..

[69] Garcia-Roger E.M., del Mar Sánchez-Montoya M., Gómez R., Suárez M.L., Vidal-Abarca M.D.R., Latron J., Rieradevall M., Prat N. (2011). Do seasonal changes in habitat features influence aquatic macroinvertebrate assemblages in perennial versus temporary Mediterranean streams?. Aquat. Sci..

[70] Munné A., Prat N. (2011). Effects of Mediterranean climate annual variability on stream biological quality assessment using macroinvertebrate communities. Ecol. Indic.

[71] Cid N., Bonada N., Carlson S.M., Grantham T.E., Gasith A., Resh V.H. (2017). High Variability is a defining Component of Mediterranean-Climate rivers and their biota. Water.

[72] Dolédec S., Tilbian J., Bonada N. (2017). Temporal variability in taxonomic and trait compositions of invertebrate assemblages in two climatic regions with contrasting flow regimes. Sci. Total Environ..

[73] Monroy S., Martínez A., López-rojo N., Pérez-calpe A.V., Basaguren A., Pozo J. (2017). Structural and functional recovery of macroinvertebrate communities and leaf litter decomposition after a marked drought: Does vegetation type matter?. Sci. Total Environ..

[74] De Girolamo A.M., Lo Porto A., Pappagallo G., Tzoraki O., Gallart F. (2014). The hydrological status concept: Application at a temporary river (Candelaro, Italy). River Res. Appl..

[75] Acuña V., Muñoz I., Giorgi A., Omella M., Sabater F., Sabater S. (2005). Drought and postdrought recovery cycles in an intermittent Mediterranean stream: Structural and functional aspects. J. N. Am. Benthol. Soc..

[76] Buffagni A., Erba S., Cazzola M., Barca E., Belfiore C. (2020). The ratio of lentic to lotic habitat features strongly affects macroinvertebrate metrics used in southern Europe for ecological status classification. Ecol. Indic.

[77] Boersma K.S., Bogan M.T., Henrichs B.A., Lytle D.A. (2014). Invertebrate assemblages of pools in arid-land streams have high functional redundancy and are resistant to severe drying. Freshw. Bio..

[78] Warfe D.M., Hardie S.A., Uytendaal A.R., Bobbi C.J., Barmuta L.A. (2014). The ecology of rivers with contrasting flow regimes: Identifying indicators for setting environmental flows. Freshw. Bio..

[79] Vander Vorste R., Mermillod-Blondin F., Hervant F., Mons R., Forcellini M., Datry T. (2016). Increased depth to the water table during river drying decreases the resilience of Gammarus pulex and alters ecosystem function. Ecohydrology.

[80] Leigh C., Datry T. (2017). Drying as a primary hydrological determinant of biodiversity in river systems: A broad-scale analysis. Ecography.

[81] Arenas-Sánchez A., Rico A., Vighi M. (2016). Effects of water scarcity and chemical pollution in aquatic ecosystems. Sci. Total Environ..

[82] Bonada N., Cañedo-Argüellez M., Gallart F., von Chiller D., Fortuño P., Latron J., Llorens P., Múrria C., Soria M., Vynioles D. (2020). Conservation and management of isolated pools in temporary rivers. Water.

[83] Bonada N., Rieradevall M., Resh V.H. (2006). Benthic macroinvertebrate assemblages and macrohabitat connectivity in Mediterranean-climate streams of northern California. J. N. Am. Benthol. Soc..

[84] Kelso J.E., Entrekin S.A. (2018). Intermittent and perennial macroinvertebrate communities had similar richness but differed in species trait composition depending on flow duration. Hydrobiologia.

[85] Bogan M., Boersma K., Litle D. (2015). resistance and resilience of invertebrate communities to seasonal and supraseasonal drought in arid-land headwater streams. Freshw. Bio..

[86] Leigh C., Bonada N., Boulton A.J., Hugueny B., Larned S.T., Vander Vorste R., Datry T. (2016). Invertebrate assemblage responses and the dual roles of resistance and resilience to drying in intermittent rivers. Aquat. Sci..

[87] Oertli B., Joye D.A., Castella E., Cambin D., Lachavanne J. (2002). Does size matter? The relationship between pond area and biodiversity. Biol. Conserv..

[88] Vander Vorste R., Corti R., Sagouis A., Datry T. (2015). Invertebrate communities in gravel-bed, braided rivers are highly resilient to flow intermittence. Freshw. Sci..

[89] Lobera G., Pardo I., Garcia L., Garcia C. (2019). Disentangling spatio-temporal drivers influencing benthic communities in temporary streams. Aquat. Sci..

[90] Lobera G., Pardo I. (2021). Response of resources and consumers to experimental flow pulses in a temporary Mediterranean stream. Sci. Total Environ..

[91] Feminella J.W. (1996). Flow Permanence Comparison of benthic macroinvertebrate assemblages in small streams along a gradient of flow permanence. J. N. Am. Benthol. Soc..

[92] Chadd R.P., England J.A., Constable D., Dunbar M.J., Extence C.A., Leeming D.J., Murray-Bligh J.A., Wood P.-J. (2017). An index to track the ecological effects of drought development and recovery on riverine invertebrate communities. Ecol. Indic.

[93] Drummond L.R., Mcintosh A.R., Larned S.T. (2015). Invertebrate community dynamics and insect emergence in response to pool drying in a temporary river. Freshw. Bio..

[94] Magurran A.E. (1988). Measuring Biological Diversity.

[95] Wilding N.A., White J.C., Chadd R.P., House A., Wood P.J. (2018). The influence of flow permanence and drying pattern on macroinvertebrate biomonitoring tools used in the assessment of riverine ecosystems. Ecol. Indic.

[96] Datry T., Corti R., Heino J., Hugueny B., Rolls R.J., Ruhí A., Datry T., Bonada N. (2017). Habitat fragmentation and metapopulation, metacommunity, and metaecosystem dynamics in intermittent rivers and ephemeral streams. Intermittent Rivers and Ephemeral Streams.

[97] Klausmeyer K.R., Shaw M.R. (2009). Climate change, habitat loss, protected areas and the climate adaptation potential of species in Mediterranean ecosystems worldwide. PLoS ONE.

[98] Hershkovitz Y., Gasith A. (2013). Resistance, resilience, and community dynamics in mediterranean-climate streams. Hydrobiologia.

[99] Nikolaidis N.P., Demetropoulou L., Froebrich J., Jacobs C., Gallart F., Prat N., Lo Porto A., Campana C., Papadoulakis V., Skoulikidis N. (2013). Towards sustainable management of Mediterranean river basins: Policy recommendations on management aspects of temporary streams. Water Policy.

[100] Garcia C., Gibbins C.N., Pardo I., Batalla R.J. (2017). Long term flow change threatens invertebrate diversity in temporary streams: Evidence from an island. Sci. Total Environ..

[101] White J.C., House A., Punchard N., Hannah D.M., Wilding N.A., Wood P.J. (2018). Macroinvertebrate community responses to hydrological controls and groundwater abstraction effects across intermittent and perennial headwater streams. Sci. Total Environ..

[102] Bonada N., Dolédec S., Statzner B. (2007). Taxonomic and biological trait differences of stream macroinvertebrate communities between Mediterranean and temperate regions: Implications for future climatic scenarios. Glob. Chang. Biol..

[103] Myers N., Mittermeier R.A., Mittermeier C.G., da Fonseca G.A.B., Kent J. (2000). Biodiversity hotspots for conservation priorities. Nature.

[104] Boulton A.J. (2014). Conservation of ephemeral streams and their ecosystem services: What are we missing?. Aquat. Conserv. Mar. Freshw. Ecosyst..

[105] Smeti E., von Schiller D., Karaouzas I., Laschou S., Vardakas L., Sabater S., Tornés E., Monllor-Alcaraz L.S., Guillem-Argiles N., Martinez E. (2019). Multiple stressor effects on biodiversity and ecosystems functioning in a Mediterranean temporary river. Sci. Total Environ..

[106] Fuentealba C., Figueroa R., Morrone J.J. (2010). Análisis de endemismo de moluscos dulceacuícolas de Chile. Rev. Chil. Hist. Nat..

