# Supplementary material for: Hydrological, Environmental and Taxonomical Heterogeneity during the Transition from Drying to Flowing Conditions in a Mediterranean Intermittent River
Source: Biology (Basel). 2021 Apr 9;10(4):316. doi: 10.3390/biology10040316 (PMC8068964; doi:10.3390/biology10040316)
Supplement: Supplementary file 1 [file biology-10-00316-s001.pdf]

Table S1. Indicators of Hydrologic Alteration- IHA in the Lonquén River.

**Period of Analysis: 1986–2015 (30 years)**	
**Normalization Factor**	**1**
Mean annual flow	13.25
Non-Normalized Mean Flow	13.25
Annual C. V.	3.54
Flow predictability	0.38
Constancy/predictability	0.48
% of floods in 60d period	0.39
Flood-free season	38

**Indicators of Hydrologic Alteration-IHA**	**IHA Analysis**		**IHA Analysis (Excluding Zero Flow Days)**	
**Parameter Group #1**	**Medians**	**Coeff. of Disp.**	**Medians**	**Coeff. of Disp.**
October	1.7	1.8	1.7	1.6
November	0.4	3.3	0.6	1.5
December	0.0	0.0	0.3	1.7
January	0.0	0.0	0.1	4.3
February	0.0	0.0	0.2	4.3
March	0.0	0.0	0.2	3.7
April	0.0	0.0	0.3	2.8
May	0.0	0.0	0.4	5.6
June	1.8	14.2	5.0	5.1
July	10.8	2.4	10.9	2.4
August	17.2	1.3	16.7	1.0
September	6.0	1.3	5.9	1.4
**Parameter Group #2**	**Medians**	**Coeff. of Disp.**	**Medians**	**Coeff. of Disp.**
1-day minimum	0.044	4.517		
3-day minimum	0.04592	5.385		
7-day minimum	0.04766	5.532		
30-day minimum	0.06867	6.356		
90-day minimum	0.1309	3.889		
1-day maximum	304	1.537		
3-day maximum	243.5	1.534		
7-day maximum	179.8	1.443		
30-day maximum	83.75	1.423		
90-day maximum	48.58	1.11		
Number of zero days	0	0		
Base flow index	0.00464	5.606		
**Parameter Group #5**	**Medians**	**Coeff. of Disp.**		
Rise rate	0.01569	14		
Fall rate	−0.3	−2.217		
Number of reversals	39	0.3205		

**The Environmental Flow Components (EFC)**		
**EFC Parameters**	**Medians**	**Coeff. of Disp.**
Extreme low peak	0.015	2.383
Extreme low duration	24	2.75
Extreme low timing	342	0.3989
Extreme low freq.	1	1
High flow peak	19.15	2.033

High flow duration	5.5	2.205
High flow timing	213	0.1202
High flow frequency	4	0.875
High flow rise rate	9.824	1.121
High flow fall rate	-3.363	-0.907
Small Flood peak	444.5	0.5124
Small Flood duration	80	0.8344
Small Flood timing	197	0.1769
Small Flood freq.	0	0
Small Flood rise rate	20.44	3.434
Small Flood fall rate	-15.12	-0.8313
Large flood peak	901	0.263
Large flood duration	45	0.9556
Large flood timing	194	0.1694
Large flood freq.	0	0
Large flood rise rate	225.2	1.13
Large flood fall rate	-20.86	-2.82

EFC low flow threshold	
EFC high flow threshold	5.58
EFC extreme low flow threshold	0.05805

EFC small flood minimum peak flow	304
EFC large flood minimum peak flow	671.8

Table S2. Percentage of the aquatic states frequency of the Lonquén River.

	OCT	NOV	DEC	JAN	FEB	MAR	APR	MAY	JUN	JUL	AUG	SEP
**Hyporheic**	0.0%	3.2%	48.4%	77.4%	80.6%	80.6%	74.2%	35.5%	12.9%	0.0%	0.0%	0.0%
**Arheic**	0.0%	16.1%	29.0%	22.6%	19.4%	19.4%	16.1%	19.4%	0.0%	0.0%	0.0%	0.0%
**Oligorheic**	9.7%	16.1%	16.1%	0.0%	0.0%	0.0%	3.2%	9.7%	0.0%	0.0%	0.0%	0.0%
**Eurheic**	90.3%	64.5%	6.5%	0.0%	0.0%	0.0%	6.5%	29.0%	74.2%	87.1%	93.5%	100%
**Hyperrheic**	0.0%	0.0%	0.0%	0.0%	0.0%	0.0%	0.0%	6.5%	12.9%	12.9%	6.5%	0.0%

Table S3. Aquatic invertebrate taxa (Family and taxon) recorded in the Lonquén River, according to aquatic states and sampling sites (PE: Puente Esmeralda; RM: Rincomavida; BA: Buenos Aires; TR: Trehuaco).

			**Oligorheic-Drying ORD**				**Arheic ARH**				**Oligorheic-Rewetting ORR**				**Eurheic EUR**			
Phylum	***Family***	***Taxa***	**PE**	**RM**	**BA**	**TR**	**PE**	**RM**	**BA**	**TR**	**PE**	**RM**	**BA**	**TR**	**PE**	**RM**	**BA**	**TR**
Cnidaria	Hydridae	*Hydra* sp.	0	1	0	0	0	0	1	0	0	0	0	0	0	0	0	0
Mollusca	Hyriidae	*Diplodon chilensis*	0	0	0	0	0	7	0	0	0	0	0	0	0	0	0	0
	Hydrobiidae	*Littoridina cumingii*	0		0	0	0	1	0	0	0	0	15	0	0	0	0	0
	Chilinidae	*Chilina dombeyana*	13	0	0	0	1	0	0	0	0	0	0	0	2	0	0	0
	Physidae	*Physa chilensis*	11	47	36	852	1	3	5	317	4	0	21	7	8	4	2	1
	Planorbidae	*Biomphalaria chilensis*	0	1	0	0	0	0	0	0	0	0	0	0	0	0	0	0
	Ancylidae	*Gundlachia gayana*	29	5	5	0	0	1	0	0	1	0	5	0	1	1	0	0
Annelida	Naiididae	*Nais* sp.	385	353	130	15336	5	0	101	38	0	8	0	0	0	39	49	0
	Lumbriculidae	*Lumbricus* sp.	0	85	0	130	2	62	76	90	222	128	160	124	0	26	2	3
	Glossiphoniidae	*Helobdella* sp.	1	0	0	0	0	0	0	0	0	0	1	0	0	0	0	0
Arthropoda	Hydracarina	*Hydracarina*	208	856	612	101	0	0	76	0	17	1	2	6	0	1	0	3
	Daphniidae	*Daphnia ambigua*	139	504	279	19	2	5	102	0	7	0	243	0	0	1	0	0
	Bosminidae	*Eubosmina hagmani*	0	0	0	0	0	0	0	0	0	59	4169	49	0	0	0	0
	Calanoida	*Calanoida*	7	152	0	0	0	0	0	0	1	4	122	10	0	0	0	1
	Cyclopoida	*Cyclopoida*	0	1	3	26	2	84	81	7	319	294	1362	224	0	8	0	0
	Harpacticoida	*Harpacticoida*	541	432	127	241	2	13	71	3	0	21	0	28	0	0	0	1
	Cyprididae	*Herpetocypris reptans*	91	683	309	274	3	15	57	244	41	84	59	28	15	5	0	4
		Cypridopsis vidua	108	266	214	282	2	66	8	56	684	536	18	96	5	31	1	2
	Hyalellidae	*Hyalella costera*	34	29	36	66	0	0	0	1	0	1	1	0	0	0	1	3
	Aeglidae	*Aegla* sp.	0	0	1	0	0	1	0	0	0	0	0	0	0	0	0	0
	Parastacidae	*Samastacus spinifrons*	0	0	0	0	0	1	1	0	0	0	0	0	0	0	0	0
	Collembola	*Collembola*	0	0	0	1	0	0	0	0	15	1	0	10	1	2	5	1
	Caenidae	*Caenis* sp.	402	1662	561	5	0	1	0	1	0	0	1	0	73	24	34	49
	Leptophlebiidae	*Leptophlebiidae*	0	0	0	0	0	0	0	0	0	0	0	0	0	1	8	29
	Baetidae	*Andesiops* sp.	70	189	1068	2690	0	2	0	2	0	0	31	0	2	26	18	63
	Aeshnidae	*Aeshnidae*	62	16	17	0	0	1	0	28	0	0	2	0	0	0	0	0
	Coenagrionidae	*Coenagrionidae*	156	16	5	0	0	0	0	2	0	1	6	0	0	0	0	0
	Petaluridae	*Petaluridae*	0	1	0	0	0	0	0	0	0	0	0	0	0	0	0	0
	Libelullidae	*Libelullidae*	0	0	0	0	0	0	0	0	0	0	0	0	0	0	0	0

Gripopterygidae	*Gripopterygidae*	0	0	0	0	0	0	0	0	0	2	0	0	1	10	10	53
Corixidae	*Sigara* sp.	126	57	47	32	0	1	0	1	1	0	5	0	3	1	0	0
Notonectidae	*Notonecta* sp.	3	0	0	0	0	0	1	0	0	0	0	0	0	0	0	0
Belastomatidae	*Belostoma elegans*	131	22	0	0	0	0	0	0	13	0	0	0	0	0	0	0
Heteroceridae	*Heterocerus* sp.	0	23	0	0	0	0	0	0	0	0	0	0	0	0	0	0
Hydrophilidae	*Tropisternus lateralis*	1	2	0	1	0	5	1	7	0	1	1	0	0	0	0	0
Dytiscidae	*Ranthus* sp.	0	0	1	28	0	4	0	8	1	0	11	1	18	5	0	9
	Neoporus sp.	0	0	1	1	0	0	0	0	0	0	0	0	1	0	0	0
Elmidae	*Elmidae*	0	0	0	0	0	0	0	0	0	0	1	0	0	0	0	0
Hydraenidae	*Hydraena* sp.	0	0	0	0	0	0	0	0	1	0	0	0	0	0	0	0
Helephoridae	*Helephorus* sp.	1	6	0	0	0	0	0	0	0	0	0	0	0	0	0	0
Staphylinidae	*Xantholinus* sp.	0	0	0	0	0	0	0	0	0	0	0	0	0	0	2	3
Leptoceridae	*Oecetis* sp.	1	45	13	0	0	0	0	0	0	0	0	0	0	0	0	0
Hydropsychidae	*Smicridea* sp.	0	1	1	1	0	0	0	0	0	0	0	0	0	0	0	0
Hydroptilidae	*Ochrotrichia* sp.	0	0	3	0	0	0	0	0	0	0	0	0	0	0	0	0
	Oxyethira sp.	1	0	1	16	0	0	0	0	0	0	0	0	0	0	0	0
Chironomidae	*Chironomidae*	710	2352	2890	12595	11	82	124	135	201	461	152	231	311	276	256	685
Simuliidae	*Simulium* sp.	0	0	651	0	0	0	0	0	0	0	0	0	0	31	7	2
Ceratopogonidae	*Ceratopogonidae*	177	271	61	5	5	12	34	36	30	28	8	41	5	5	5	0
Limoniidae	*Limonia* sp.	0	14	1	4	0	0	0	0	0	0	0	0	10	2	1	1
Ephidridae	*Ephydra riparia*	1	7	1	204	0	0	0	2	0	0	0	0	3	1	1	3
	Scatella sp.	0	0	0	0	0	1	0	0	1	0	0	0	0	2	15	10
Chamaemyiidae	*Leucopis* sp.	0	6	0	1	0	1	0	1	0	0	0	0	1	0	0	0
Tabanidae	*Tabanus* sp.	2	1	0	1	0	0	0	0	0	0	0	0	6	3	10	2
